# The Effect of Vitamin D_3_ Supplementation on the Incidence of Diagnosed Dementia Among Healthy Older Adults—The Finnish Vitamin D Trial

**DOI:** 10.1093/gerona/glaf077

**Published:** 2025-04-17

**Authors:** Eija Lönnroos, Maija Ylilauri, Christel Lamberg-Allardt, JoAnn E Manson, Tarja Nurmi, Matti Uusitupa, Ari Voutilainen, Sari Hantunen, Tomi-Pekka Tuomainen, Jyrki K Virtanen

**Affiliations:** Institute of Public Health and Clinical Nutrition, University of Eastern Finland, Kuopio, Finland; Institute of Public Health and Clinical Nutrition, University of Eastern Finland, Kuopio, Finland; Department of Food and Nutrition, University of Helsinki, Helsinki, Finland; Department of Medicine, Brigham and Women’s Hospital, Harvard Medical School, Boston, Massachusetts, USA; Department of Epidemiology, Harvard T.H. Chan School of Public Health, Boston, Massachusetts, USA; Institute of Public Health and Clinical Nutrition, University of Eastern Finland, Kuopio, Finland; Institute of Public Health and Clinical Nutrition, University of Eastern Finland, Kuopio, Finland; Institute of Public Health and Clinical Nutrition, University of Eastern Finland, Kuopio, Finland; Institute of Public Health and Clinical Nutrition, University of Eastern Finland, Kuopio, Finland; Institute of Public Health and Clinical Nutrition, University of Eastern Finland, Kuopio, Finland; Institute of Public Health and Clinical Nutrition, University of Eastern Finland, Kuopio, Finland; (Medical Sciences Section)

**Keywords:** Cognitive decline, General population, Randomized controlled trial

## Abstract

**Background:**

Some short-term vitamin D supplementation trials suggest benefits on cognitive performance, but apart from observational studies, there is little evidence whether long-term vitamin D supplementation can prevent development of dementia. We investigated whether vitamin D_3_ supplementation could affect the incidence of diagnosed dementia in a generally healthy population.

**Methods:**

The study included 2 492 participants from the Finnish Vitamin D Trial, free of diagnosed dementia at baseline. They were randomized to placebo, 1 600 IU/d, or 3 200 IU/d of vitamin D_3_ arm for up to 5 years. Incident diagnoses of dementia were obtained from the national care registries.

**Results:**

The mean age of the participants at baseline was 68.2 years and 42.8% were female. During the mean follow-up of 4.2 years, 18 participants in the placebo arm, 14 participants in the 1 600 IU/d arm (compared to placebo, hazard ratio [HR] = 0.77, 95% confidence interval [CI]: 0.38–1.55), and 13 participants in the 3 200 IU/d arm (HR = 0.72, 95% CI: 0.35–1.48) were diagnosed with dementia. Of the diagnoses, 29 were Alzheimer’s disease, without statistically significant differences in the event rates between the 3 arms. Age, sex, or body mass index did not modify the effects. In the subgroup of 550 participants, the mean baseline serum 25-hydroxyvitamin D concentration was 74.8 nmol/L. After 12 months, the mean concentrations were 73.0, 99.7, and 120.4 nmol/L in the placebo, 1 600 IU/d, and 3 200 IU/d arms, respectively.

**Conclusions:**

Five-year, medium-dose or high-dose vitamin D_3_ supplementation did not affect the dementia incidence in this largely vitamin D-sufficient older population.

**Clinical Trial Registry Number:**

ClinicalTrials.gov: NCT01463813, https://clinicaltrials.gov/ct2/show/NCT01463813

Population aging is a global phenomenon (1). By the year 2050, the share of the world’s population aged 60 years or over has been projected to be 22% and 80% of older people living in low- and middle-income countries, respectively. Age and effects of aging, especially age-related neuropathologies, increase the risk for cognitive decline and dementia (2,3). With population aging, the global number of people with dementia will increase substantially from the present 55 million to 139 million in 2050 (4).

Dementia syndromes can be caused by a variety of diseases and conditions that affect the brain, such as Alzheimer’s disease (AD) or vitamin deficiencies (5). Dementia is characterized by a decline in cognitive abilities severe enough to impair daily and social functioning. Disability associated with dementia causes a significant burden to individuals, families, and societies and is a key driver of healthcare costs. In 2019, the estimated total costs of dementia were $23 796 per person with dementia, and the global annual costs were $1.3 trillion (6), which is approximately $550 billion higher than the annual diabetes-related care costs (7). In the United States, the estimated care cost of AD and other dementias ($345 billion) exceeds those of heart disease and stroke ($251 billion) and cancer ($240 billion) (8).

While there is at present no cure for AD or other progressive dementias, the efforts to promote brain health and prevent or delay the onset of dementia via modifiable risk factors are public health priorities (9,10). One factor that may potentially play a role in the development of cognitive impairment is vitamin D insufficiency, which is prevalent globally (11). Vitamin D acts via its physiologically active form, 1,25(OH)_2_D, which is a steroid hormone that interacts with vitamin D receptors. These receptors are ubiquitous in different tissues and cell types, and also many regions of the brain that are associated with cognition have vitamin D receptors (12). Indeed, in observational studies, poor vitamin D status has been associated with worse performance on cognitive tests (13) and with increased risk of developing dementia and AD (14). In human experimental studies, vitamin D supplementation has had a beneficial effect on some domains of cognition (15), and mechanistic studies in animal models and different cell cultures have provided potential mechanisms that could explain the neuroprotective properties of vitamin D (16). Even though the exact molecular mechanisms are not fully elucidated, vitamin D seems to be involved in anti-inflammatory, antioxidant, antiapoptotic, and anticholinesterase activity, and it has a neuroprotective role and an immunomodulatory effect (16). Many of these effects are conducted in an interaction between vitamin D and the vitamin D receptor via genomic and nongenomic pathways (16). For example, vitamin D may protect against amyloid-β accumulation, a key factor in early neurodegeneration, and reduce tau phosphorylation and nitric oxide synthase activity that are induced by amyloid-β (16–18).

However, although some short-term clinical trials suggest potential benefits of vitamin D supplementation on performance in tests that assess cognitive capacity (15), less is known about the effects of supplementation on the incidence of diagnosed dementia. In some observational studies, users of vitamin D supplements have had a lower risk for developing dementia during the follow-up (19,20), but some studies have reported even an increased risk among vitamin D supplement users (21). However, the findings from observational studies need to be interpreted cautiously because of potential issues with confounding and reverse causation.

Long-term clinical trials with incident dementia as an outcome are needed to investigate the causality of vitamin D supplementation on the development of dementia, but currently such research data are scarce. Therefore, we investigated the effects of 5-year vitamin D supplementation (1 600 or 3 200 IU/d vs placebo) on the incidence of diagnosed dementia among generally healthy, older adults from the Finnish Vitamin D Trial (FIND).

## Method

### Study Population

The FIND study was conducted between September 2012 and October 2018. It was a double-blind, placebo-controlled randomized trial designed to investigate the effects of 5-year vitamin D_3_ supplementation on the incidence of chronic diseases among generally healthy men and women from a general population in Finland (22). The primary endpoints of the trial were cardiovascular disease (CVD) and cancer. Memory disorders were a prespecified ancillary outcome.

The inclusion criteria were: male participants aged ≥60 years and postmenopausal female participants aged ≥65 years without a history of CVD or cancer (except nonmelanoma skin cancer). The higher minimum age in women reflects the occurrence of many chronic diseases later in life in women compared to men. The exclusion criteria were: history of kidney stones, renal failure or dialysis, hypercalcemia, hypo- or hyperparathyroidism, severe liver disease (cirrhosis), or sarcoidosis or other granulomatous diseases such as active chronic tuberculosis or Wegener’s granulomatosis; and use of vitamin D >800 IU/d or calcium >1 200 mg/d from all supplemental sources combined (or if taking, not willing to decrease or forego such use during the trial).

**Figure 1.**
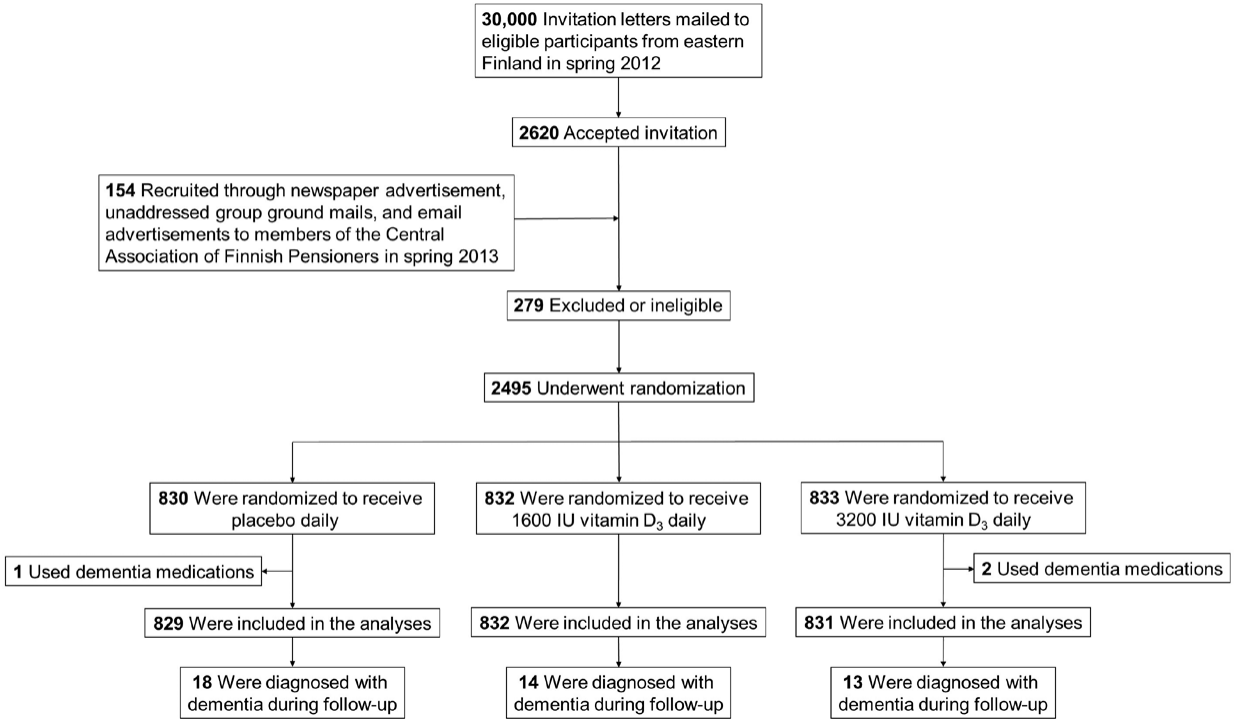
Participant flow chart.

The original goal was to recruit 18 000 participants for the trial, an equal number of men and women. With this number of participants, the study would have had adequate power to detect moderate differences in the incidence of diseases over 5 years. However, the recruitment process was much more challenging than expected and yielded only a total of 2 495 participants. They were randomized into 3 groups: (i) 1 600 IU/d of vitamin D_3_, (ii) 3 200 IU/d of vitamin D_3_, or (iii) placebo (Figure 1). Randomization into the 3 groups was done by sex-stratified simple randomization, with a 1:1:1 ratio, based on computerized random number generation. The randomization was conducted by a statistician from outside of the study group. The 1 600 IU/d dose was designed to be as close as possible (with the over-the-counter supplements available in Finland in early 2010s) to the 2 000 IU/d dose used in the VITamin D and Omega-3 TriaL (VITAL) (23) to help in comparing the study findings. The 3 200 IU/d dose was selected to be as large as possible while not exceeding (with the 800 IU/d of personal vitamin D supplementation that the participants were allowed to take during the trial) the 4 000 IU/d no-observed-adverse-effect level (NOAEL). For the trial, over-the-counter vitamin D supplements were not used, but instead the study pills were purchased from Galena Pharma Ltd (Kuopio, Finland). One pill contained either 0 IU, 1 600 IU, or 3 200 IU of vitamin D_3_. The pills were annually either mailed to the participants or given at study visits. Double-anonymized was maintained throughout the study.

A random subgroup of 551 participants underwent more detailed baseline examinations. Of those, 512, 533, and 496 participants took part in the in-person clinic-based follow-up visits at 6, 12, and 24 months, respectively (22).

Participants completed questionnaires 5 times throughout the trial: at baseline, after 12, 24, and 36 months, and at the conclusion of the 60-month study period (22). The baseline, 36-month, and 60-month questionnaires included a validated 142-item food-frequency questionnaire. Additionally, the final questionnaire included an inquiry regarding adherence to the study protocol, asking participants, “How much of the study pills did you take during the study?” Response options ranged from “<50%” to “100% or almost 100%.” Body mass index (BMI) calculations were derived from the weight and height data provided in the baseline questionnaire.

Serum 25(OH)D_3_ concentrations were measured using high-performance liquid chromatography (24). Samples were collected from the subgroup at baseline (*n* = 550), and after 6 (*n* = 511) and 12 (*n* = 532) months (22).

All participants were volunteer adults who were entitled to withdraw from the study at any time without explanation. All participants signed a written informed consent. The appropriate study approvals were obtained from the Ethics committee of the Kuopio University Hospital (#30/2010) and from the Finnish Institute for Health and Welfare.

### Assessment of Dementia Diagnosis

Detecting and screening of cognitive symptoms are usually conducted in primary health care in Finland. The diagnostic evaluation of dementing disorders has been centralized to outpatient memory clinics operating both in primary and specialized health care. In the FIND study, dementia incidents were identified through national registers. The International Classification of Diseases, 10th Revision codes F00-F03 and G30-G31 were considered to indicate dementia. The study participants who received a dementia diagnosis were retrieved by means of their personal identification codes (previously known as social security numbers) from 3 national registers: Care Register for Health Care (25) and Register of Primary Health Care Visits (26), both maintained by the Finnish Institute for Health and Welfare (data agreement: THL/523/5.05.00/2020), and Causes of Death register maintained by Statistics Finland (data agreement: TK/1007/07.03.00/2022) (27). The Care Register for Health Care covers the data on discharges from inpatient care (primary, secondary, and tertiary care hospitals) and outpatient visits in specialized health care. The Register of Primary Health Care Visits covers all primary care outpatient visits. Moreover, the study participants who reported the use of drugs for dementia (Anatomical Therapeutic Chemical [ATC] codes N06D) in the FIND study questionnaires were classified as diagnosed with dementia. Only one of the 21 participants who reported the use of dementia drugs during the follow-up had no corresponding ICD-10 code of dementia in the national registers.

### Statistical Analysis

Study participants who reported the use of drugs for dementia at baseline (*n* = 3) were excluded from the statistical analyses. The Cox proportional hazards model was used to predict the hazard of dementia, adjusted for age and sex. The log rank (Mantel–Cox) test was used to detect trends over the vitamin D supplementation levels (placebo, 1 600 IU/d, and 3 200 IU/d). As per prespecified protocol, the 2 supplementation arms were compared to the placebo arm. In the additional, nonprespecified analyses, we combined the 2 vitamin D arms and compared that to the placebo arm. We also excluded from the analyses the events that occurred during the first 2 years of follow-up and included the events that occurred during the post-supplementation period to investigate potential latent effects of supplementation. Unadjusted multiplicative interactions between the intervention and subgroups (age, sex, BMI) were also modeled. In the analyses, 2 follow-up scenarios with respect to the use of registers were applied: the 5-year supplementation period and the extended period until December 31, 2021. In the first scenario, the follow-up ended if the study participant had a diagnosis of dementia, met one of the FIND study primary endpoints (major CVD or cancer), withdrew, or perished during the 5-year supplementation period. In the second scenario, the extended follow-up period lasted until a diagnosis of dementia, withdrawing from the study, death, or the end of follow-up period. IBM SPSS version 29 served as the statistical platform (IBM SPSS Statistics, Armonk, NY). Two-sided *p* <.05 indicated statistical significance. The *p* values were not adjusted for multiple comparisons.

## Results

The mean age of the 2 492 study participants was 68.2 (*SD* 4.5) years, and most (75.1%) were ≥65 years old. There were no significant differences in the baseline characteristics between the 3 study arms (Table 1). In the subgroup of 550 participants with available data on serum 25(OH)D, the mean baseline concentration was 74.8 nmol/L (*SD* 18.2) (*p* for difference between the arms = .72), with concentrations <50 nmol/L observed in 9.1% of the participants and concentrations of ≥75 nmol/L in 50.0% of the participants (*p*-difference = .93). After 12 months, the mean concentrations were 73.0 nmol/L (*SD* 17.8) in the placebo arm, 99.7 nmol/L (*SD* 21.1) in the 1 600 IU/d arm, and 120.4 nmol/L (*SD* 21.8) in the 3 200 IU/d arm (*p*-difference < .001). Among the 1 608 participants who filled the final study questionnaire at 60 months and answered the question about adherence, 74.8% (*n* = 1 203, *p*-difference between the arms = .42) reported using all or almost all study pills and 95.3% (*n* = 1 532, *p*-difference = .82) reported using at least 80% of the pills during the 5-year supplementation period.

**Table 1.** Baseline Characteristics of the Participants

Characteristic	Overall (*n* = 2 492)	Placebo (*n* = 829)	1 600 IU/d of Vitamin D_3_ (*n* = 832)	3 200 IU/d of Vitamin D_3_ (*n* = 831)
Female sex, *n* (%)	1 069 (42.8)	372 (44.8)	349 (41.9)	348 (41.8)
Age, mean (*SD*), y	68.2 (4.5)	68.2 (4.5)	68.1 (4.5)	68.3 (4.4)
Age group, *n* (%)				
60–64 y*	620 (24.9)	202 (24.4)	209 (25.1)	209 (25.2)
65–69 y	1 088 (43.7)	373 (45.0)	358 (43.0)	357 (43.0)
70–74 y	588 (23.6)	185 (22.3)	203 (24.4)	200 (24.1)
≥75 y	196 (7.9)	69 (8.3)	62 (7.5)	65 (7.8)
Employment status, *n* (%)	*n* = 2 466	*n* = 822	*n* = 824	*n* = 820
Full time work	201 (8.2)	76 (9.2)	61 (7.4)	64 (7.8)
Part-time work	95 (3.9)	27 (3.3)	38 (4.6)	30 (3.7)
Unemployed	61 (2.5)	14 (1.7)	25 (3.0)	22 (2.7)
Retired	2 094 (84.9)	702 (85.4)	692 (84.0)	700 (85.4)
Not working for other reasons	15 (0.6)	3 (0.4)	8 (1.0)	4 (0.5)
Leisure-time physical activity, mean (*SD*), h/wk^†^				
Light	13.1 (10.8) (*n* = 2 169)	13.1 (10.9) (*n* = 711)	13.3 (11.2) (*n* = 721)	12.8 (10.4) (*n* = 737)
Heavy	5.8 (6.5) (*n* = 1 599)	5.6 (6.3) (*n* = 525)	5.8 (6.6) (*n* = 532)	5.9 (6.6) (*n* = 542)
Smoking regularly^‡^, *n* (%)	883 (35.7) (*n* = 2 474)	291 (35.3) (*n* = 824)	300 (36.5) (*n* = 823)	292 (35.3) (*n* = 827)
At least high school diploma, *n* (%)	415 (16.7) (*n* = 2 480)	134 (16.2) (*n* = 826)	149 (18.0) (*n* = 827)	132 (16.0) (*n* = 827)
Married, *n* (%)	1 847 (74.7) (*n* = 2 473)	619 (75.2) (*n* = 823)	606 (73.5) (*n* = 825)	622 (75.4) (*n* = 825)
Body mass index, mean (*SD*), kg/m^2^	27.1 (4.3) (*n* = 2 488)	27.2 (4.3) (*n* = 828)	27.1 (4.3) (*n* = 832)	26.9 (4.3) (*n* = 831)
Alcohol intake, mean (*SD*), g/d	7 (13) (*n* = 2 461)	8 (16) (*n* = 824)	7 (11) (*n* = 818)	7 (11) (*n* = 822)
Vitamin D intake from diet, mean (*SD*), IU/d	428 (312) (*n* = 2 461)	414 (260) (*n* = 823)	456 (382) (*n* = 818)	420 (282) (*n* = 820)
Use of own vitamin D supplements, *n* (%)				
Not at all	1 667 (66.9)	534 (64.4)	566 (68.0)	567 (68.2)
200−400 IU/d	361 (14.5)	133 (16.0)	104 (12.5)	124 (14.9)
>400–<800 IU/d	74 (3.0)	27 (3.3)	26 (3.1)	21 (2.5)
800 IU/d	390 (15.6)	135 (16.3)	136 (16.3)	119 (14.3)
Self-rated health: good or excellent, *n* (%)	1 459 (59.2) (*n* = 2 466)	459 (55.9) (*n* = 821)	493 (60.3) (*n* = 818)	507 (61.3) (*n* = 827)

*Notes*:

*All are men in this age group.

^†^Light activity defined as gardening, other light outdoor activities, etc. Heavy activity defined as physical exercise that causes sweating or heavy breathing.

^‡^Smoking regularly defined as smoking almost every day during the last year.

### Incidence of Dementia During the 5-Year Vitamin D Supplementation Period

**Figure 2.**
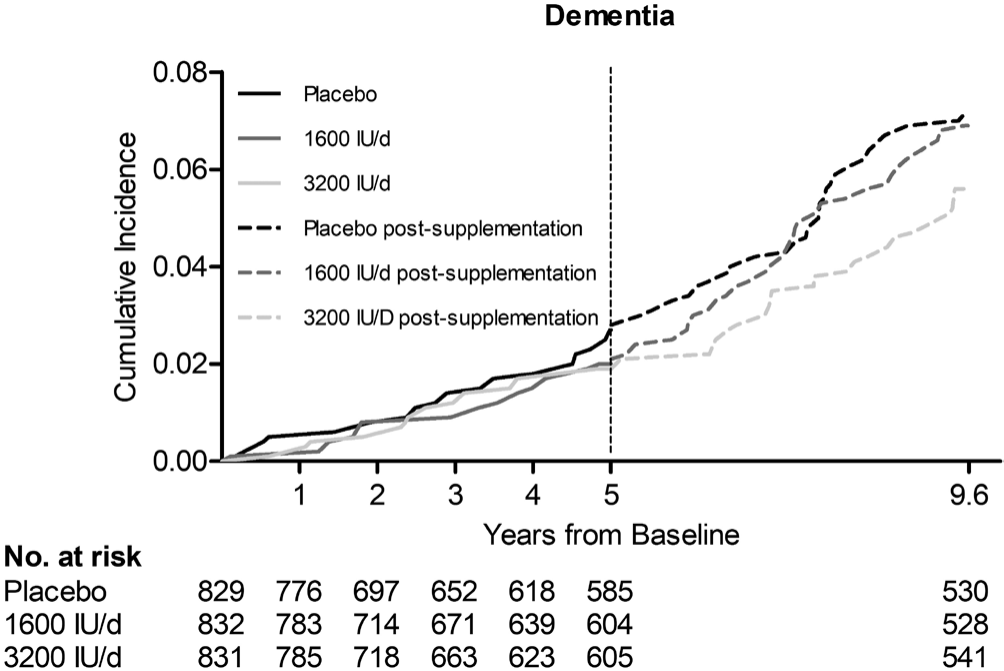
Cumulative incidence of dementia in the 3 study arms during the 5-y supplementation period and in the extended post-supplementation follow-up.

During the 5-year vitamin D supplementation period (mean follow-up 4.2 years), altogether 45 study participants were diagnosed with dementia. Their age at the time of dementia diagnosis ranged from 65 to 89 years with the mean (median) age of 77 (78) years. AD was the most common cause of dementia (*n* = 29), and the diagnosis was received at the age of 66–89 years with the mean (median) age of 79 (78) years. The incidence of all-cause dementia per 100 person–years (PY) was the highest (0.52 [95% CI: 0.24–0.68]) in the placebo arm and the lowest (0.37 [95% CI: 0.22–0.64]) in the vitamin D 3 200 IU/d supplementation arm. Using the placebo arm as the reference, the age- and sex-adjusted hazard ratios (HRs) for all-cause dementia were not statistically significantly lower for the vitamin D 1 600 IU/d supplementation arm (HR 0.77 [95% CI: 0.38–1.55], *p* = .46), the 3 200 IU/d supplementation arm (HR 0.72 [95% CI: 0.35–1.48], *p* = .37), or the combined vitamin D arms (HR 0.75 [95% CI: 0.41–1.36], *p* = .33) (Table 2, Figure 2). Neither were statistically significant protective effects of vitamin D supplementation found when the 16 participants diagnosed with dementia within the first 2 years of the follow-up were excluded from the analyses (Table 2). The incidence of AD decreased by increasing vitamin D supplementation, but the age- and sex-adjusted HRs for AD were not statistically significantly lower compared with the placebo arm (Table 2).

**Table 2.** Incidence of Dementia During the 5-Year Supplementation Period According to Randomization Arm*

Event	Placebo (*n* = 829)	1 600 IU/d of Vitamin D_3_ (*n* = 832)	*p* Value	3 200 IU/d of Vitamin D_3_ (*n* = 831)	*p* Value	*p* Value for Trend	Combined Vitamin D Arms vs Placebo	*p* Value
PY, *n*	3451.0	3534.5		3509.6				
Events, *n*	18	14		13				
Rate per 100 PY (95% CI)	0.52 (0.33–0.83)	0.40 (0.23–0.67)		0.37 (0.22–0.64)				
Hazard ratio (95% CI)	1	0.77 (0.38–1.55)	.462	0.72 (0.35–1.48)	.374	.334	0.75 (0.41–1.36)	.337
After exclusion of 16 participants with dementia diagnosis within the first 2 y of follow-up								
PY, *n*	3446.0	3526.5		3504.9				
Events, *n*	12	8		9				
Rate per 100 PY (95% CI)	0.35 (0.20–0.61)	0.23 (0.11–0.45)		0.26 (0.13–0.49)				
Hazard ratio (95% CI)	1	0.66 (0.27–1.61)	.360	0.75 (0.32–1.78)	.516	.465	0.70 (0.34–1.48)	.353
After exclusion of 16 participants with dementia not related to Alzheimer’s disease								
PY, *n*	3436.8	3521.6		3497.1				
Events, *n*	12	9		8				
Rate per 100 PY (95% CI)	0.35 (0.20–0.61)	0.26 (0.13–0.49)		0.23 (0.11–0.46)				
Hazard ratio (95% CI)	1	0.68 (0.29–1.64)	.394	0.68 (0.28–1.65)	.389	.341	0.68 (0.32–1.43)	.308

*Notes*: PY = person–years.

*Adjusted for age and sex in the Cox proportional hazards model.

The effects of vitamin D supplementation were also examined in analyses stratified by sex, age, and BMI (Table 3). Vitamin D supplementation of 1 600 or 3 200 IU/d or their combination did not show statistically significant effects on the 5-year risk of all-cause dementia for men or women, for the participants below or above the median age of 67.4 years or by any of the BMI classes.

**Table 3.** Incidence of Dementia During the 5-Year Supplementation Period According to Subgroup and Randomization Arm

Endpoint	No. of Participants	Placebo	1 600 IU/d of Vitamin D_3_	3 200 IU/d of Vitamin D_3_	*p* Value for Trend	*p* Value for Interaction	Combined Vitamin D Arms vs Placebo	*p* Value for Interaction
Men (events, *n*)	1 424	7	7	5		.614		.347
		1	0.83 (0.29–2.39)	0.64 (0.20–2.02)	.486		0.74 (0.29–1.88)	
Women (events, *n*)	1 068	11	7	8				
		1	0.71 (0.28–1.84)	0.79 (0.32–1.95)	.548		0.75 (0.34–1.63)	
Age < median 67.4 y (events, *n*)	1 247	2	4	2		.845		.562
		1	1.96 (0.36–10.7)	1.02 (0.14–7.24)	.982		1.50 (0.30–7.43)	
Age ≥ median 67.4 y (events, *n*)	1 245	16	10	11				
		1	0.61 (0.28–1.36)	0.66 (0.31–1.42)	.252		0.64 (0.33–1.22)	
BMI < median 26.4 kg/m^2^ (events, *n*)	1 245	12	10	7		.332		.140
		1	0.80 (0.34–1.85)	0.53 (0.21–1.34)	.178		0.66 (0.31–1.38)	
BMI ≥ median 26.4 kg/m^2^ (events, *n*)	1 243	6	4	6				
		1	0.67 (0.19–2.38)	1.10 (0.35–3.42)	.921		0.88 (0.32–2.42)	
BMI < 25 kg/m^2^ (events, *n*)	849	7	8	7		.206		.075
		1	1.00 (0.36–2.77)	0.78 (0.27–2.23)	.692		0.88 (0.36–2.17)	
BMI = 25–30 kg/m^2^ (events, *n*)	1 132	8	4	4				
		1	0.50 (0.15–1.68)	0.56 (0.17–1.88)	.264		0.53 (0.20–1.43)	
BMI ≥ 30 kg/m^2^ (events, *n*)	507	3	2	2				
		1	0.84 (0.14–5.08)	0.75 (0.13–4.51)	.712		0.79 (0.18–3.56)	

*Notes*: Values are hazard ratios (95% confidence intervals) adjusted for age and sex in the Cox proportional hazards model. Four study participants did not report their weight and/or height and were excluded from the BMI subgroups. BMI = body mass index.

### Incidence of Dementia During the Extended Follow-up Period

During the extended follow-up (mean 7.7 years) until the end of the year 2021, 131 incident dementia diagnoses (most AD, *n* = 96) were observed (Supplementary Table 1). The incidence rates of all-cause dementia were 0.65, 0.62, and 0.51/100 PY for the placebo, 1 600 IU/d, and 3 200 IU/d supplementation arms, respectively. Compared with the placebo arm, neither the 1 600 IU/d (HR 0.95 [95% CI: 0.61–1.47], *p* = .81) or 3 200 IU/d (HR 0.79 [95% CI: 0.50–1.25], *p* = .32) nor the combined vitamin D arms (HR 0.87 [95% CI: 0.59–1.28], *p* = .48) showed statistically significantly decreased risk for incident dementia. Age- and sex-adjusted HRs for all-cause dementia remained statistically nonsignificant after excluding the participants diagnosed with dementia during the first 2 study years. Neither for AD was a statistically significant protective effect found in the extended follow-up (Supplementary Table 1).

In the sex-, age-, and BMI-stratified analyses (Supplementary Table 2), vitamin D supplementation had no statistically significant effects on the risk of all-cause dementia during the extended follow-up. However, age modified the association between vitamin D and dementia. Though not statistically significant, the number of events and HRs for dementia decreased by increasing vitamin D dose among older participants (≥67.4 years) but not among younger participants.

## Discussion

We conducted a randomized, placebo-controlled 5-year vitamin D supplementation trial among generally healthy Finns with median age of 67 years at baseline. To our knowledge, this was the first randomized controlled trial (RCT) that has investigated the effects of high-dose vitamin D supplementation on the dementia incidence. Compared to placebo, vitamin D supplementation of 1 600 or 3 200 IU/d did not reduce the incidence of all-cause dementia or AD during the mean 4.2-year supplementation period or in the extended mean 7.7-year follow-up.

The role of vitamin D in cognitive functioning and development of dementia has been investigated in several epidemiological studies, where low serum 25(OH)D concentrations have been linked with worse cognitive performance (13) and increased risk of dementia (14). However, observational studies can be affected by reverse causality and confounding. In the case of vitamin D, low serum 25(OH)D concentrations are commonly associated with, for example, higher adiposity, smoking, lower physical activity, and history of many chronic diseases. Therefore, in observational studies, low serum 25(OH)D concentration could be a marker for these other factors rather than a causal risk factor itself. On the other hand, there are several potential mechanisms that could explain a beneficial effect of vitamin D on cognition (16), and a recent meta-analysis of vitamin D supplementation trials suggested a small but significant positive effect on global cognition in adults, especially in vulnerable patients and those with vitamin D deficiency (15). Furthermore, a Canadian observational cohort study (19) and a UK Biobank cohort study (20) found that vitamin D supplementation might also have potential in dementia prevention. However, as observational studies, they do not provide strong evidence for causality, and the authors of both studies acknowledged that randomized placebo-controlled vitamin D supplementation trials with dose–response analyses are essential for definitive evidence. To our knowledge, there is only one other RCT in addition to our study that has investigated the effect of vitamin D supplementation on dementia incidence, but that study included also a calcium supplement and the vitamin D dose was quite low. In that study among 4 143 women from the Women’s Health Initiative who were 65 years or older and had the baseline mean serum 25(OH)D concentration of about 50 nmol/L, 400 IU/d of vitamin D_3_ and 1 000 mg/d of calcium did not affect the risk of dementia over the mean follow-up of 7.8 years.

The Lancet Commission on dementia prevention listed 14 modifiable risk factors for dementia (low educational level, hypertension, high low-density lipoprotein cholesterol concentration, hearing impairment, vision loss, smoking, obesity, depression, physical and social inactivity, diabetes, excessive alcohol consumption, traumatic brain injury, air pollution) through which nearly half of dementias worldwide could be theoretically prevented (10). The participants of the present study had many of the risk factors listed above and were a general population without a history of CVD or cancer, the primary endpoints of the FIND study. As an intervention to prevent dementia, vitamin D supplementation is appealing. It is safe, easy to implement also at a population level, and inexpensive, thus suitable also for the low- and middle-income countries. Yet, many of the single substance or single domain dementia prevention trials, including vitamin and mineral supplementations, have not shown benefits or have provided low-quality evidence only (10,28,29). Nearly all the present evidence on dementia prevention is from studies conducted in high-income countries, and the recommendations given have substantial similarities with CVD and diabetes prevention (10,29). The research interests in dementia prevention are increasingly focused on multidomain interventions with substantial variability in their target populations and contents (30). This might be a strength, as there seldom is a simple and uniform solution for problems with complex and heterogenous etiology.

A major strength of the FIND study was the randomized, double-blind and placebo-controlled design. Another strength was the use of 2 vitamin D doses that enabled the dose–response evaluations. In addition, the population-based recruitment of the participants underlies the generalizability of the findings. The combined use of the 3 national registers to identify incident cases of dementia was another strength of our study. By using the registries, losses to follow-up were minimized, especially during the post-supplementation period when the participants were no longer contacted. The accuracy of dementia diagnoses in the national registers was very good even a couple of decades ago, but the occurrence of dementia and AD were underestimated (31). After a pilot period in 2008–2009, the Register of Primary Health Care Visits was implemented and started nationwide data collection in 2011 (26). As the diagnostic evaluation of cognitive symptoms is conducted mainly in outpatient settings, the new primary care register improved the likelihood of capturing all of the diagnoses made.

One limitation of the study was that the data on the serum 25(OH)D concentrations were available only for the subcohort of participants, and that the concentrations were at a sufficient level at baseline among most participants. Thus, we could not investigate whether vitamin D supplementation could be beneficial among those with vitamin D insufficiency (serum 25(OH)D < 50 nmol/L), but a trial targeting those with vitamin D deficiency would not have been ethical due to the need for many to receive placebo for multiple years. The relatively high serum 25(OH)D concentrations at the beginning of the study (prior to randomization) most likely reflect the vitamin D food fortification programs in Finland that were implemented in 2003 and 2010, and also the increased use of vitamin D supplements (32). In the year 2000, the mean vitamin D intake from diet in both men and women in Finland was 140 IU/d, but it increased to 480 IU/d in women and to 560 IU/d in men in 2011 (32). During the same time period, the mean serum 25(OH)D concentrations increased from 48 nmol/L (19 ng/mL) to 65 nmol/L (26 ng/mL), and the prevalence of vitamin D deficiency (defined as serum 25(OH)D < 50 nmol/L) decreased from 56% to 9% (32). Thus, although the methods to assess vitamin D intakes differ between the studies and the lack of a standardized serum 25(OH)D assay method makes comparisons difficult, the dietary vitamin D intake and the mean serum 25(OH)D concentrations at baseline in the FIND study are generally similar to the population-level estimates (32). The main limitations of the study were the low number of participants and incident dementia events, which limited statistical power for both the dose–response analyses and the pooled-group analyses. Assuming an annual incidence of dementia in Finland of about 0.6% in these age groups (33,34) and a 25% reduction in the incidence in the 3 200 IU/d vitamin D arm versus the placebo arm, the trial would have needed to have about 7 125 participants per arm to have enough power to detect statistically significant differences in the incidence. With the current number of participants, the study had power to detect only a very large, about 65% annual decrease in the dementia incidence in the vitamin D arms compared to the placebo.

The fairly young age of the participants (median 67 years, 25% <65 years) can be seen both as a strength and a weakness of our study. It was possible to detect early-onset (<65 years of age) dementias, though many of them are caused by genetic variants or mutations, such as presenilin or amyloid precursor protein mutations in early-onset AD (35) or hereditary or sporadic gene mutations in frontotemporal dementia (36). However, such events were not observed in our study: none of the participants was diagnosed with dementia under the age of 65 years. The incidence of dementia increases sharply with age and peaks among the oldest old (34). In high-income countries, the incidence doubles with every 5.8-year increase of age, from 3.9 per 1 000 PY at age of 60–64 years to 125/1 000 at age of 90+. In our study, the treatment and follow-up were relatively short for an investigation of dementia, which limited the number of events. Many dementing diseases, including AD, progress slowly and have long latent and preclinical phases (5). To prevent or delay the clinical onset of a dementing disorder probably requires a long-term intervention as well. Although it is possible to develop a lifelong register-based follow-up for incident dementia, a lifelong vitamin D RCT would be challenging. Finally, the FIND study population included only White participants from Finland, so we were not able to investigate possible differences by ethnicity or race, which limits the generalizability of the findings. The VITAL study assessed the effect of vitamin D_3_ supplementation (2 000 IU daily) on memory and global cognition over 2–3 years and did not observe associations in the overall cohort; however, vitamin D had benefits for cognition among the African American participants (37).

## Conclusion

In conclusion, in comparison to placebo, medium-dose (1 600 IU/d) or high-dose (3 200 IU/d) vitamin D_3_ supplementation for 5 years did not significantly affect the incidence of clinically diagnosed dementia among generally healthy, largely vitamin D-sufficient and, with respect to the risk of dementia, relatively young Finnish participants. However, the study did not have adequate power to detect weak or average effects of vitamin D supplementation, so such effects cannot be ruled out with this study. Although short-term trials suggest some benefit for cognitive performance with vitamin D supplementation, there is paucity of research evidence for whether this would also manifest in the prevention of dementia. Therefore, additional evidence from vitamin D supplementation trials with diagnosed dementing diseases as the outcome is needed, before definite conclusions regarding the impact of vitamin D supplementation on the prevention of cognitive decline can be drawn. Such trials would need to include a large number of subjects, have a long follow-up, and preferably include multiple vitamin D doses for evaluation of dose–response. Although inclusion of mainly participants with known vitamin D deficiency would be preferable, for ethical reasons, such trials could not have a placebo group with zero supplemental vitamin D intake. One strategy in these situations could be to have the control group supplement with a small dose of vitamin D, for example, 400 IU/d. However, it might be challenging to recruit a large number of vitamin D-deficient participants for such a trial, especially in countries with national vitamin D food fortification programs or where the use of vitamin D supplements is common, and it is possible that 400 IU/d supplementation could modify risk in the “placebo” group. Until evidence from these kinds of trials is available (if it ever will be), the results from the FIND study and from other similar long-term vitamin D supplementation trials can be pooled, preferably in individual participant data meta-analyses, in order to increase statistical power to detect low-to-moderate effects of vitamin D supplementation and to help identify population subgroups that might especially benefit from supplementation.

## Supplementary Material

glaf077_suppl_Supplementary_Tables

## Data Availability

The data will not be openly available because they contain sensitive personal information of the participants that cannot be completely anonymized. However, the analytical code used for the current study can be made available upon reasonable request, and the data are open for potential research collaboration by contacting the corresponding author.
