# Supplementary material for: The Effect of Vitamin D3 Supplementation on the Incidence of Diagnosed Dementia Among Healthy Older Adults—The Finnish Vitamin D Trial
Source: J Gerontol A Biol Sci Med Sci. 2025 Apr 17;80(7):glaf077. doi: 10.1093/gerona/glaf077 (PMC12149738; doi:10.1093/gerona/glaf077)
Supplement: glaf077_suppl_Supplementary_Tables [file glaf077_suppl_supplementary_tables.docx]

SUPPLEMENTAL MATERIAL

SUPPLEMENTAL TABLE 1. Incidence of dementia during the 5-year supplementation period and extended post supplementation follow-up period until the end of year 2021 according to randomization arm^1^

| Event | Placebo (n=829) | 1600 IU/day of vitamin D_3_ (n=832) | *P* value | 3200 IU/day of vitamin D_3_ (n=831) | *P* value | *P* value for trend | Combined vitamin D arms vs. placebo | *P* value |
| --- | --- | --- | --- | --- | --- | --- | --- | --- |
| PY, *n* | 6346.5 | 6481.7 |  | 6465.9 |  |  |  |  |
| Events, *n* | 48 | 46 |  | 37 |  |  |  |  |
| Rate per 100 PY (95% CI) | 0.76 (0.57−1.00) | 0.71 (0.53−0.95) |  | 0.57 (0.41−0.79) |  |  |  |  |
| Hazard ratio (95% CI) | 1 | 0.93 (0.62−1.40) | 0.738 | 0.76 (0.50−1.17) | 0.213 | 0.201 | 0.85 (0.59−1.21) | 0.362 |
| *After exclusion of 17^2^ participants with dementia diagnosis within the first 2 years of follow-up* | | | | | | | | |
| PY, *n* | 6341.1 | 6473.8 |  | 6461.2 |  |  |  |  |
| Events, *n* | 41 | 40 |  | 33 |  |  |  |  |
| Rate per 100 PY (95% CI) | 0.65 (0.48−0.88) | 0.62 (0.45−0.84) |  | 0.51 (0.36−0.72) |  |  |  |  |
| Hazard ratio (95% CI) | 1 | 0.95 (0.61−1.47) | 0.815 | 0.79 (0.50−1.25) | 0.321 | 0.307 | 0.87 (0.59−1.28) | 0.480 |
| *After exclusion of 35 participants with dementia not related to Alzheimer’s disease* | | | | | | | | |
| PY, *n* | 6288.6 | 6416.7 |  | 6411.9 |  |  |  |  |
| Events, *n* | 36 | 34 |  | 26 |  |  |  |  |
| Rate per 100 PY (95% CI) | 0.57 (0.41−0.79) | 0.53 (0.38−0.74) |  | 0.41 (0.28−0.60) |  |  |  |  |
| Hazard ratio (95% CI) | 1 | 0.92 (0.57−1.47) | 0.722 | 0.73 (0.44−1.21) | 0.218 | 0.180 | 0.82 (0.54−1.25) | 0.360 |

PY, person-years.

^1^Adjusted for age and sex in the Cox proportional-hazards model.

^2^In these analyses the number of participants with dementia diagnosis within the first two years of follow-up is one higher than in the analyses restricted to the 5-year supplementation period (Table 2). This is because one participant had a dementia diagnosis on day 180, but before that had a diagnosis of a primary study endpoint on day 112 of the follow-up and was therefore excluded from the study. However, that participant is included in these intention-to-treat analyses with the extended follow-up.

SUPPLEMENTAL TABLE 2. Incidence of dementia during the 5-year supplementation period and extended post supplementation follow-up period until the end of year 2021 according to subgroup and randomization arm

| Endpoint | No. of  participants | Placebo | 1600 IU/day of vitamin D_3_ | 3200 IU/day of vitamin D_3_ | *P* value  for trend | *P* value for interaction | Combined vitamin D arms vs. placebo | *P* value for interaction |
| --- | --- | --- | --- | --- | --- | --- | --- | --- |
| Men (events, *n*) | 1424 | 21 | 26 | 15 |  | 0.312 |  | 0.135 |
|  |  | 1 | 1.04 (0.58−1.86) | 0.64 (0.33−1.24) | 0.243 |  | 0.84 (0.50−1.43) |  |
| Women (events, *n*) | 1068 | 27 | 20 | 22 |  |  |  |  |
|  |  | 1 | 0.85 (0.48−1.52) | 0.88 (0.50−1.55) | 0.571 |  | 0.87 (0.54−1.41) |  |
| Age <median 67.4 y (events, *n*) | 1247 | 7 | 10 | 6 |  | **0.048** |  | **0.025** |
|  |  | 1 | 1.41 (0.54−3.71) | 0.86 (0.29−2.57) | 0.824 |  | 1.14 (0.47−2.77) |  |
| Age ≥median 67.4 y (events, *n*) | 1245 | 41 | 36 | 31 |  |  |  |  |
|  |  | 1 | 0.85 (0.54−1.33) | 0.71 (0.45−1.13) | 0.143 |  | 0.78 (0.53−1.15) |  |
| BMI <median 26.4 kg/m^2^ (events, *n*) | 1245 | 28 | 29 | 21 |  | 0.220 |  | 0.102 |
|  |  | 1 | 0.96 (0.57−1.62) | 0.67 (0.38−1.17) | 0.175 |  | 0.81 (0.51−1.29) |  |
| BMI ≥median 26.4 kg/m^2^ (events, *n*) | 1243 | 20 | 17 | 16 |  |  |  |  |
|  |  | 1 | 0.86 (0.45−1.64) | 0.89 (0.46−1.71) | 0.902 |  | 0.87 (0.50−1.52) |  |
| BMI <25 kg/m^2^ (events, *n*) | 849 | 21 | 21 | 17 |  | 0.192 |  | 0.100 |
|  |  | 1 | 0.87 (0.47−1.59) | 0.64 (0.34−1.21) | 0.209 |  | 0.75 (0.44−1.27) |  |
| BMI 25-30 kg/m^2^ (events, *n*) | 1132 | 18 | 17 | 14 |  |  |  |  |
|  |  | 1 | 0.90 (0.46−1.75) | 0.84 (0.42−1.70) | 0.586 |  | 0.87 (0.49−1.57) |  |
| BMI ≥30 kg/m^2^ (events, *n*) | 507 | 9 | 8 | 6 |  |  |  |  |
|  |  | 1 | 1.04 (0.40−2.72) | 0.74 (0.26−2.07) | 0.567 |  | 0.88 (0.38−2.05) |  |

Values are hazard ratios (95% confidence intervals) adjusted for age and sex in the Cox proportional-hazards model. Four study participants did not report their weight and/or height and were excluded from the BMI subgroups.
